# Methanandamide diminish the *Porphyromonas gingivalis* lipopolysaccharide induced response in human periodontal ligament cells

**DOI:** 10.1186/s12903-020-01087-6

**Published:** 2020-04-15

**Authors:** Fengqiu Zhang, Burcu Özdemir, Phuong Quynh Nguyen, Oleh Andrukhov, Xiaohui Rausch-Fan

**Affiliations:** 1 Department of Periodontology, Capital Medical University School of Stomatology, Beijing, China; 2 Division of Conservative Dentistry and Periodontology, University Clinic of Dentistry, Medical University of Vienna, Sensengasse 2a, 1090 Vienna, Austria; 3 Department of Periodontology, Faculty of Dentistry, Gazi University, Ankara, Turkey

**Keywords:** Methanandamide, Cell proliferation, Cytokines, Chemokines, Periodontal ligament

## Abstract

**Background:**

The endocannabinoid system is involved in the regulation of periodontal tissue homeostasis. Synthetic cannabinoid methanandamide (Meth-AEA) has improved stability and affinity to cannabinoid receptors compared to its endogenous analog anandamide. In the present study, we investigated the effect of methanandamide on the production of pro-inflammatory mediators in primary human periodontal ligament cells (hPdLCs).

**Methods:**

hPdLCs were treated with Meth-AEA for 24 h, and the resulting production of interleukin (IL)-6, IL-8, and monocyte chemotactic protein (MCP)-1 was measured in the absence or the presence of *Porphyromonas gingivalis* lipopolysaccharide (LPS). Additionally, the effect of Meth-AEA on the proliferation/viability of hPdLCs was measured by the MTT method.

**Results:**

Methanandamide at a concentration of 10 μM significantly inhibited *P. gingivalis* LPS induced production of IL-6, IL-8, and MCP-1. Basal production of IL-6 and IL-8 was slightly enhanced by 10 μM Meth-AEA. No effect of Meth-AEA on the basal production of MCP-1 was observed. Meth-AEA in concentrations up to 10 μM did not affect the proliferation/viability of hPdLCs, but significantly inhibited it at a concentration of 30 μM.

**Conclusion:**

Our study suggests that the inflammatory response in periodontal ligament cells could be influenced by the activation of the cannabinoid system, which might be potentially involved in the progression of periodontal disease.

## Background

Periodontitis is a biofilm-induced chronic inflammatory disease, which affects teeth supporting structures, including the gingival tissue, periodontal ligament, and alveolar bone [1]. Clinical signs of periodontitis are gingival inflammation, periodontal pocket formation, periodontal tissue destruction, and, in advanced cases, periodontitis might lead to alveolar bone resorption and tooth loss [2]. Gram-negative anaerobic bacteria such as *Porphyromonas gingivalis* (*P. gingivalis*) is thought to be one of the primary etiological agents of periodontitis [3, 4]. *P. gingivalis* possesses multiple virulence factors that could either induce periodontal tissue inflammation or subvert the host immune system [4, 5]. Lipopolysaccharide (LPS) is one of the most important virulence factors of *P. gingivalis* [6, 7].

The endocannabinoid (EC) system consists of endocannabinoids and cannabinoid receptor proteins. Endocannabinoids are a family of endogenous lipid neurotransmitter which activates cannabinoid receptors. Several endocannabinoids have been discovered, the most characterized ones are the anandamide (AEA) and 2-arachidonoylglycerol (2-AG) [8], which might be produced by various cells like osteoblasts, osteoclasts, and endothelial cells [9, 10]. Cannabinoid receptors belong to a transmembrane G-protein coupled receptor family. The primary endocannabinoid receptors cannabinoid receptor type 1 (CB1) and cannabinoid receptor type 2 (CB2) are expressed in various cells and tissues and particularly in dental tissues [11]. The EC system is thought to regulate several brain processes; however, actual studies suggest its involvement in the regulation of bone physiology and immune response [12, 13].

Since the endocannabinoid system is involved in the regulation of bone formation and immune response, several recent studies investigated the mutual role of this system in the homeostasis of periodontal tissue under healthy and inflammatory conditions. Both AEA and 2-AG are detectable in gingival crevicular fluid, and their level seems to be increased in periodontally diseased individuals [14, 15]. There are some controversies about the changes in the expression of CB1 and CB2 receptors in periodontitis. One study suggests that the expression of CB1 and CB2 is upregulated under pathological conditions [14]. In contrast, another study shows that bacterial inflammation results in the decrease of CB1 expression and the increase of CB2 expression [16]. Activation of the EC system promotes survival and neuronal differentiation of periodontal ligament stem cells [17]. However, the exact role of the EC system in the progression of periodontal disease remains unknown.

Several experimental studies investigate the mutual role of the EC system in the homeostasis of periodontal tissue. In vitro studies show that anandamide stimulates the proliferation of human gingival fibroblasts [15] and diminishes cytokine production by these cells in response to stimulation with *P. gingivalis* LPS [14]. Our recent study shows that AEA and 2-AG have a different effect on *P. gingivalis* LPS induced production of interleukin (IL)-6, IL-8, and monocyte chemoattractant protein (MCP)-1 [18]. Mainly, *P. gingivalis* LPS induced response was inhibited by AEA and enhanced by 2-AG [18]. An in vivo study using the ligature periodontitis model in rats shows that the local application of AEA decreases the content of tumor necrosis factor-alpha and IL-1β in gingival tissue [19]. The effect of AEA was abolished by the simultaneous application of CB1 and CB2 inhibitors [19]. A recent study suggests that CB1 receptor might regulate osteogenic differentiation of periodontal ligament cells [20].

One of the significant problems of application of EC, and particularly AEA, in research, is their low aqueous stability, which might doubt the quality of obtained results [21]. This problem can be solved by the development of synthetic analogs of ECs [22]. Methanandamide (Meth-AEA), a synthetic analog of AEA, has a four-fold higher affinity to cannabinoid receptor than AEA itself and additionally exists a high resistance to enzymatic hydrolysis [23]. In comparison with AEA, Meth-AEA is suggested to be more selective for the CB1 receptor and less selective for the CB2 receptor [24]. Compared to ECs, the information about the effect of Meth-AEA on periodontal tissue is very limited. Only one report investigated the effect of topical application of Meth-AEA in a LPS induced periodontitis model in rats to date [25]. This study shows that Meth-AEA significantly diminishes alveolar bone loss in this periodontitis model. However, the ability of Meth-AEA to influence the inflammatory response in human cells of the periodontium is still unknown. Therefore, in the present study, we investigated the effect of Meth-AEA on the basal and *P. gingivalis* LPS induced production of some pro-inflammatory mediators by primary human periodontal ligament cells (hPdLCs).

## Methods

### Cell culture and reagents

hPdLCs were isolated from periodontal ligament tissue obtained from wisdom molars extracted for orthodontic reasons in healthy individuals similar to the method described earlier [26, 27]. All donors were systematically healthy, aged from 18 to 22 y.o. Periodontal ligament tissue was scraped from the teeth root surface with a scalpel, cut into small pieces and placed into Dulbecco’s modified Eagle’s medium (DMEM), supplemented with 10% fetal bovine serum (FBS), streptomycin (50 μg/ml) and penicillin (100 U/ml) under humidified air atmosphere of 5% CO_2_ at 37 °C. Outgrowing cells were collected and further grown in DMEM medium. hPdLCs between the third and sixth passages were used in the experiments.

Commercially available standard LPS from *P. gingivalis* (Invivogene, San Diego, CA, USA), (R)-(+)- Meth-AEA (Tocris Bristol, UK), and human soluble CD14 (Sigma, St. Louis, MO, USA) were used in the present study.

### Cell proliferation/viability assay

Cell proliferation/viability was measured by the MTT method, as described in our previous study [18]. hPdLCs were seeded in 24 well plates at a density of 2 × 10^4^ cells in 500 μL of DMEM supplemented with 10% FBS. After 24 h, the media were replaced with DMEM supplemented with 1% FBS and containing meth-AEA at concentrations of 0.03, 0.1, 0.3, 1, 3, 10, 30 μM. The hPdLCs were treated with different meth-AEA concentrations for 24 h, and untreated cells were used as a control. After treatment with Meth-AEA, 100 μl of MTT reagent (3-(4,5-dimethylthiazol-2-yl)-2,5-diphenyl-tetrazolium bromide, Sigma, St. Louis, MO, USA) were added to each well and plates were incubated at 37 °C for 2 h. After incubation, media were discarded, 500 μl of dimethylsulfoxide were added into each well to solve formed formazan crystals, and OD_550_ values were measured on a microplate reader (Molecular Devices, Sunnyvale, CA, USA). Cell proliferation/viability experiments were repeated at least three times for each donor with hPdLCs isolated from five different donors.

### Effect of meth-AEA on the production of IL-6, IL-8, and MCP-1 by hPdLCs

The hPdLCs were seeded in 24 well plate at a density of 5 × 10^4^ cells in 500 μL of DMEM supplemented with 10% FBS. After 24 h, the media were replaced with DMEM supplemented with 1% FBS and containing Meth-AEA at concentrations of 0.1, 1, 10 μM. The hPdLCs were treated with different Meth-AEA concentrations for 24 h, and untreated cells were used as a control. In some experiments, hPdLCs treatment was performed in the presence of 1 μg/ml of *P. gingivalis* LPS and 0.25 μg/ml soluble CD14 (sCD14). As shown by our recent study, sCD14 enhances the response of periodontal ligament cells to bacterial LPS [28] After 24 h stimulation, the expression of IL-6, IL-8, and MCP-1 in hPdLCs was measured by real-time PCR. The content of the corresponding protein in conditioned media was assayed by ELISA similarly to the methods described previously [18, 29].

Isolation of mRNA from hPdLCs, subsequent transcription to cDNA, and amplification were performed using commercially available TaqMan Gene Expression Cells-to-CT kit (Ambion/Applied Biosystems, Foster City, CA, USA), which provides excellent accuracy and superior sensitivity of gene-expression analysis [30]. qPCR was performed on an ABI StepOnePlus device (Applied Biosystems, Foster City, CA, USA) in paired reactions using the Taqman gene expression assays with following ID numbers (all from Applied Biosystems, Foster City, CA, USA): IL-6, Hs00985639_m1; IL-8, Hs00174103_m1; MCP-1, Hs00234140_m1; β2-microglobulin, Hs99999907_m1. Real-time PCR reactions were performed in triplicate in 96-well plates using the following thermocycling conditions: 95 °C for 10 min; 40 cycles, each for 15 s at 95 °C and 60 °C for 1 min. The point at which the PCR product was first detected above a fixed threshold (cycle threshold, Ct), was determined for each sample. Changes in the expression of target genes were calculated using the 2^-ΔΔCt^ method [31], where ΔΔCt = (C_t_^target^-C_t_^β2-microglobulin^)_sample_-(C_t_^target^-C_t_^β2-microglobulin^)_control_, taking an untreated sample as a control.

Content of IL-6, IL-8, and MCP-1 proteins in conditioned media was measured by commercially available ELISA Ready-Set-Go kits (eBioscience, San Diego, CA, USA) according to manufacturer’s instruction. Each measurement was performed in duplicates. For measurement of IL-6 and MCP-1, samples were not diluted, whereas, for the measurements of IL-8, samples were diluted 1:10.

### Statistical analysis

The normal distribution of all data was tested with the Kolmogorov-Smirnov test. After confirming normal distribution, the statistical differences between different groups were analyzed by one-way analysis of variance (ANOVA) for repeated measures followed by t-test. Statistical analyses were performed using the statistical program SPSS 21.0 (SPSS, Chicago, IL, USA). Curve fitting and regression analysis were performed with Microsoft Excel (Redmond, WA, USA). Data are expressed as mean ± S.E.M. of five different donors. Differences were considered to be statistically significant at *p* < 0.05.

## Results

### Effect of meth-AEA on proliferation/viability of hPdLCs

**Fig. 1:**
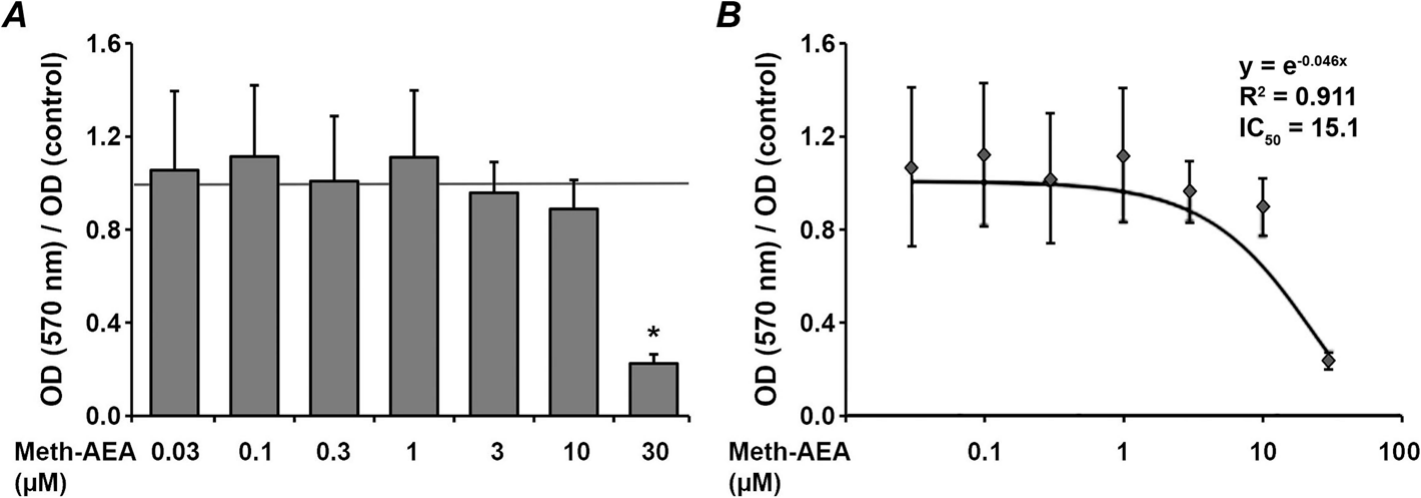
Effect of Meth-AEA on proliferation/viability of hPdLCs. hPdLCs were stimulated by different Meth-AEA concentrations for 24 h and the proliferation/viability was measured by the MTT method. **a** – OD values measured at 570 nm upon stimulation with Meth-AEA are normalized to OD values measured in the control group (ratio = 1, shown as horizontal grey line). **b** – curve fitting and regression model. Data are shown as the mean ± s.e.m. of 5 different donors. * means significantly different vs. control group (*p* < 0.05)

The effect of Meth-AEA on the proliferation/viability of hPdLCs after 24 h of stimulation is shown in Fig. 1. It can be seen that Meth-AEA at the concentrations ranging from 0.03–10 μM exerted no statistically significant effect on the proliferation/viability of hPdLCs (Fig. 1a, *p* > 0.05, ANOVA, post-hoc t-test). Treatment with meth-AEA at a concentration of 30 μM induced a significant decrease in hPdLCs proliferation/viability (Fig. 1a, *p* < 0.001, ANOVA, post-hoc t-test). The fitting curve with the regression analysis is presented in Fig. 1b.

### Effect of meth-AEA on the gene expression of IL-6, IL-8, and MCP-1 in hPdLCs

**Fig. 2:**
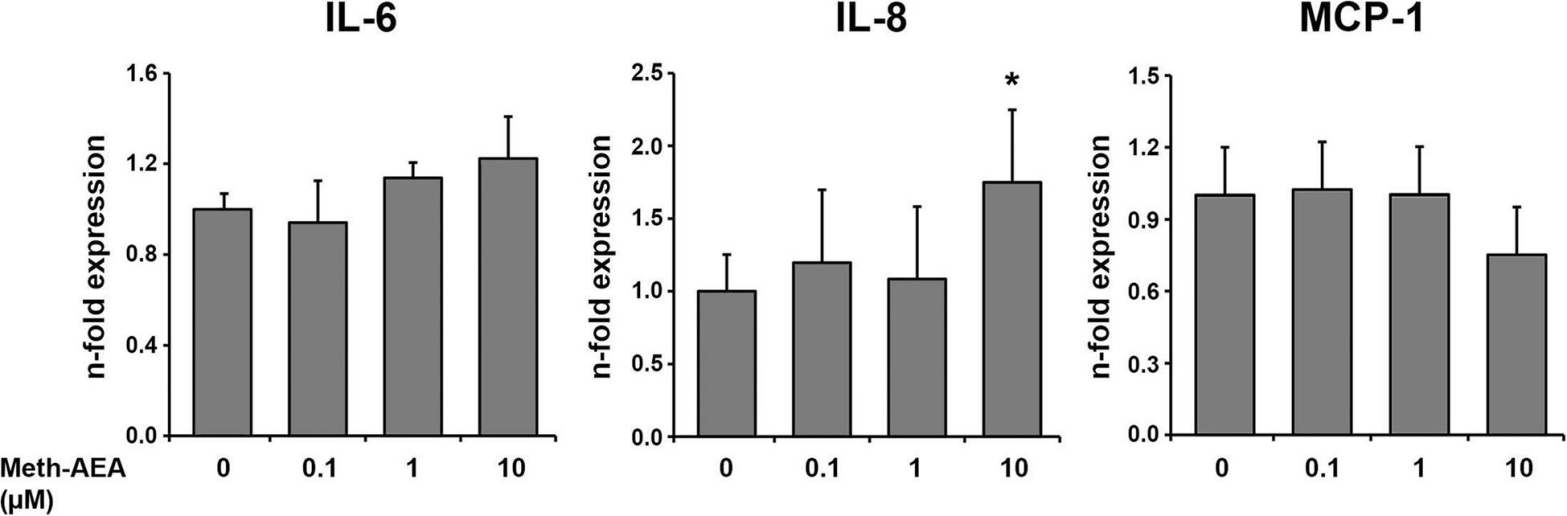
Effect of Meth-AEA on the gene expression of IL-6, IL-8, and MCP-1 in hPdLCs. Gene expression of IL-6, IL-8, and MCP-1 was measured in hPdLCs upon 24 h stimulation with different concentrations of Meth-AEA using qPCR method. Y-axes show n-fold expression levels (2^-ΔΔCt^ values) of target in relation to non-stimulated control (*n* = 1). Data are shown as mean ± s.e.m. of 5 different donors. * means significantly different vs. control group (*p* < 0.05)

Figure 2 shows the effect of Meth-AEA (0.1–10 μM) on the gene expression levels of IL-6, IL-8, and MCP-1. No significant effect of Meth-AEA in all tested concentrations on the gene expression levels of IL-6 and MCP-1 was observed. Gene expression level of IL-8 was not significantly changed by Meth-AEA in concentrations of 0.1–1 μM and was significantly increased by Meth-AEA in concentration of 10 μM.

### Effect of meth-AEA on the protein production of IL-6, IL-8, and MCP-1 by hPdLCs

**Fig. 3:**
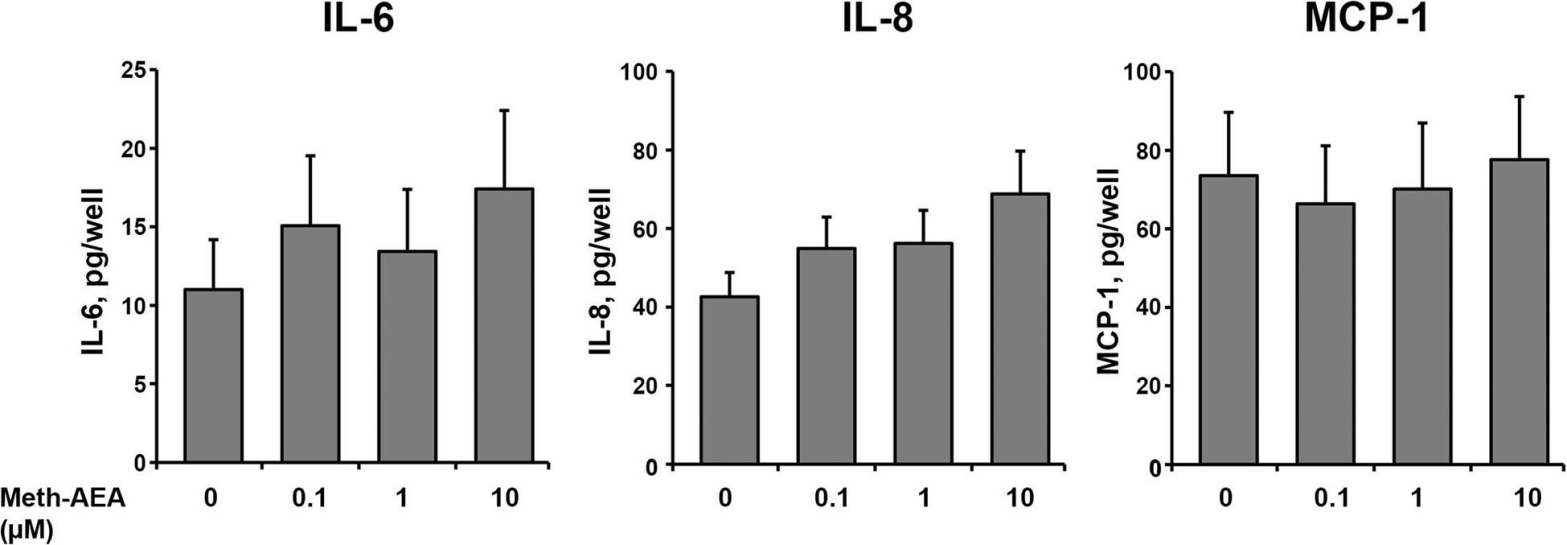
Effect of Meth-AEA on the production of IL-6, IL-8, and MCP-1 by hPdLCs. The content of IL-6, IL-8, and MCP-1 in conditioned media was measured after 24 h stimulation with different concentrations of Meth-AEA by commercially available ELISA. Data are shown as mean ± s.e.m. of 5 different donors

The effect of Meth-AEA on the production of IL-6, IL-8, and MCP-1 proteins by hPdLCs is shown in Fig. 3. As can be seen, no significant effect of Meth-AEA in concentrations of 0.1–10 μM on the production of all investigated proteins by hPdLCs was observed. Noteworthy, the content of IL-6 and IL-8 in conditioned media was increased by about 55–60% upon stimulation with 10 μM Meth-AEA, but this effect did not reach statistical significance (*p* = 0.069 for IL-6; *p* = 0.051 for IL-8).

### Effect of meth-AEA on P. gingivalis LPS induced gene expression of IL-6, IL-8, and MCP-1 in hPdLCs

**Fig. 4:**
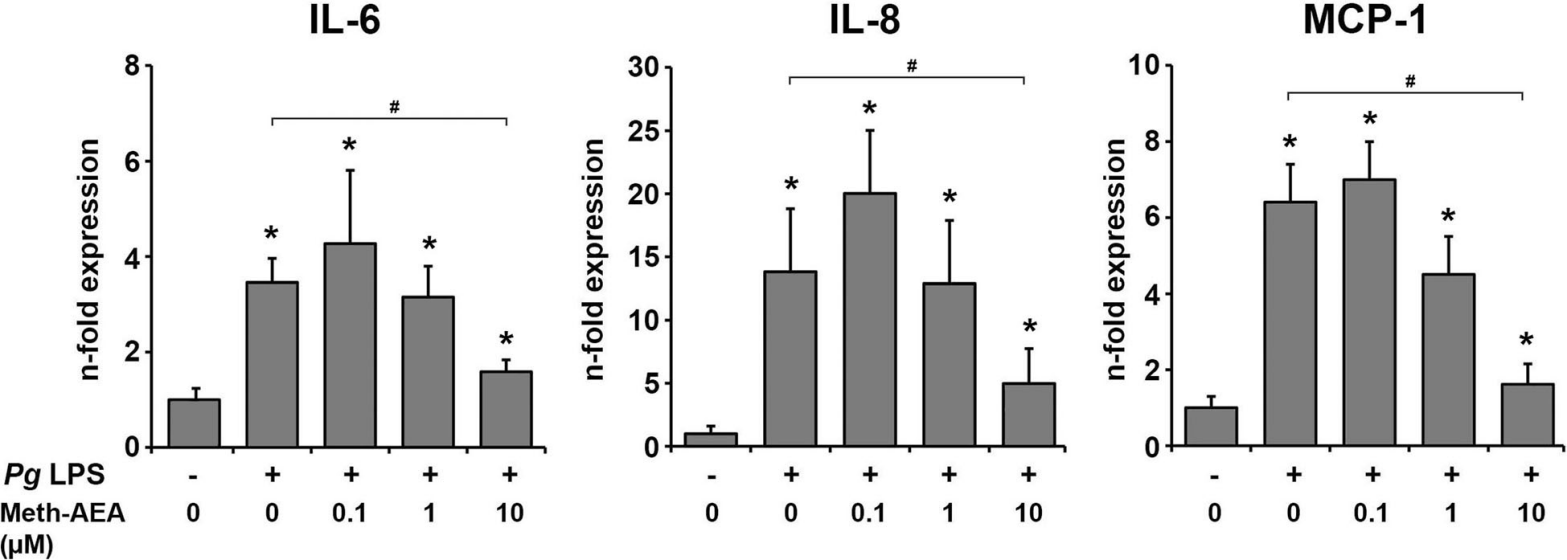
Effect of Meth-AEA on *P. gingivalis* LPS induced gene expression of IL-6, IL-8, and MCP-1 in hPdLCs. Gene expression of IL-6, IL-8, and MCP-1 was measured in hPdLCs after 24 h stimulation with *P. gingivalis* LPS and different concentrations of Meth-AEA using qPCR method. Y-axes show n-fold expression levels (2^-ΔΔCt^ values) of target in relation to non-stimulated control (n = 1). Data are shown as mean ± s.e.m. of 5 different donors. * means significantly different vs. control group (*p* < 0.05). # means significantly different vs. *P. gingivalis* LPS group (*p* < 0.05)

The effect of Meth-AEA in concentrations of 0.1–10 μM on *P. gingivalis* LPS induced gene expression of IL-6, IL-8, and MCP-1 in hPdLCs is shown in Fig. 4. *P. gingivalis* LPS induced a significant increase in the gene expression levels of all pro-inflammatory mediators. *P. gingivalis* LPS induced gene expression levels of IL-6, IL-8, and MCP-1 were significantly decreased by 10 μM Meth-AEA and were not affected by Meth-AEA in lower concentrations.

### Effect of meth-AEA on P. gingivalis LPS induced production of IL-6, IL-8, and MCP-1 in hPdLCs

**Fig. 5:**
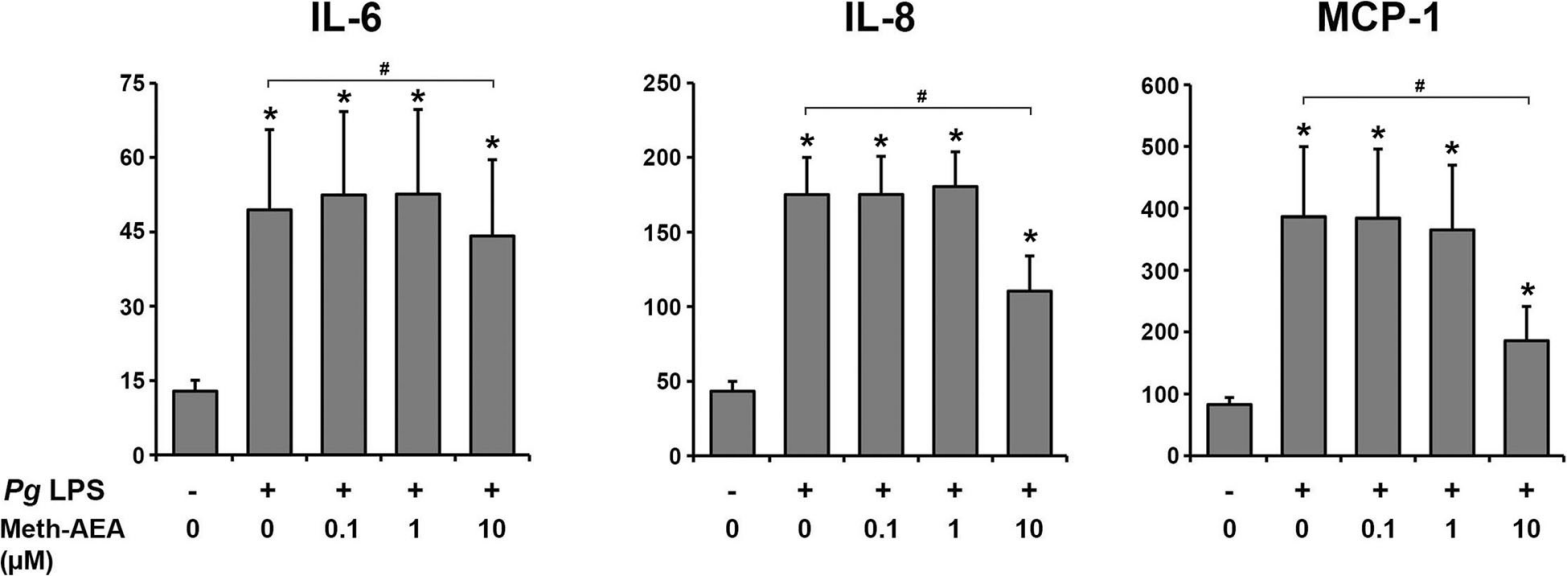
Effect of Meth-AEA on *P. gingivalis* LPS induced production of IL-6, IL-8, and MCP-1 by hPdLCs. The content of IL-6, IL-8, and MCP-1 in conditioned media was measured after 24 h stimulation with *P. gingivalis* LPS and different concentrations of Meth-AEA by commercially available ELISA. Data are shown as mean ± s.e.m. of 5 different donors. * means significantly different vs. control group (*p* < 0.05). # means significantly different vs. *P. gingivalis* LPS group (*p* < 0.05)

The content of IL-6, IL-8, and MCP-1 in conditioned media of hPdLCs upon stimulation with *P. gingivalis* LPS and different concentrations of Meth-AEA is shown in Fig. 5. *P. gingivalis* LPS significantly increased the content of all three proteins in the conditioned media. Meth-AEA at a concentration of 10 μM significantly diminished *P. gingivalis* LPS induced production of IL-8 and MCP-1 by hPdLCs, whereas lower Meth-AEA concentration had no significant effect. *P. gingivalis* LPS induced IL-6 production by hPdLCs was not significantly affected by Meth-AEA in any tested concentration.

## Discussion

Meth-AEA is a highly stable synthetic analog of AEA and, therefore, is widely used in the research on the cannabinoid system [22, 23]. In the present study, we investigated for the first time the effect of Meth-AEA on the proliferation/viability and inflammatory response in primary hPdLCs in order to further clarify the potential role of the EC system in periodontitis. hPdLCs play an essential role in the maintenance of periodontal tissue homeostasis and possess the immunomodulatory properties [32, 33]. The inflammatory response was assessed by measuring the production of IL-6,- IL-8, and MCP-1, which are involved in the progression of periodontal disease [34–36]. We have focused on these pro-inflammatory mediators because their production in hPdLCs is substantially increased by bacterial LPS [28, 37], whereas the production of other cytokines like IL-1β or tumor necrosis factor α by hPdLCs in response to LPS stimulation is rather low [38].

The proliferation/viability of hPdLCs was not significantly affected by Meth-AEA in concentrations up to 10 μM and was inhibited in a concentration of 30 μM. The effect of Meth-AEA on hPdLCs’ proliferation/viability differs from that observed for AEA. In our previous study, we found that AEA slightly stimulates the proliferation/viability of hPdLCs [18]. The exact reason for this discrepancy is not apparent, but it can be due to the different stability of Meth-AEA and AEA in aqueous solutions. Our data on Meth-AEA are in line with a study on prostate cancer cell line showing that Meth-AEA inhibits the proliferation of these cells [39].

Under resting conditions, Meth-AEA in a concentration of 10 μM significantly increased the gene expression level of IL-8. Moreover, a similar concentration of Meth-AEA induced a small, albeit non-significant increase in the production of IL-6 and IL-8 protein. In agreement with this finding, a study on the prostate cancer cell line also describes the stimulation of IL-6 production by Meth-AEA [39]. Interestingly, our previous study shows that IL-6 production by hPdLCs is slightly increased by 2-AG and not influenced by AEA [18]. Under inflammatory conditions, Meth-AEA at a concentration of 10 μM induced a significant decrease of *P. gingivalis* LPS induced production of IL-6, IL-8, and MCP-1. This effect of Meth-AEA was similar to that of AEA observed in our previous study [18]. The fact that the production of pro-inflammatory mediators is slightly stimulated by Meth-AEA under resting conditions and inhibited under inflammatory conditions suggests a multifaceted role of cannabinoid in the homeostasis of periodontal tissue. An increase in the production of IL-8 by Meth-AEA under resting conditions could be crucial for the basal activity of the innate immune system, which can, in turn, play an important role in controlling the bacteria growth. Under inflammatory conditions, Meth-AEA can potentially diminish the production of pro-inflammatory mediators by host cells and thus contribute to the protection of host tissues from collateral damages by an excessive inflammatory response.

There are several signaling pathways, which could be potentially involved in the biological effects of Meth-AEA. A biochemical study shows that Meth-AEA exhibits a high affinity for the CB1 receptor, whereas its affinity for the CB2 receptor is rather low [24]. Notably, this study reports that the affinity of Meth-AEA is about 20 nM for CB1 and about 900 nM for the CB2 receptor. In our study, the effects of Met-AEA were observed only for the concentration of 10 μM, whereas no significant effect was observed for Meth-AEA concentrations up to 1 μM. Thus, it seems that the effects of Meth-AEA could also be contributed to the activation of the CB2 receptor.

Interestingly, one study shows that Meth-AEA induces IL-6 secretion by prostate cancer cells, and this effect is inhibited by CB2 receptor antagonist SR144528 and not by CB1 receptor antagonist rimonabant [39]. An anti-inflammatory role of CB2 receptor activation in periodontitis is recently confirmed by a study of LPS-induced periodontitis in rats [40]. Here, the topical application of CB2 receptor agonist HU-308 significantly attenuates the bone loss and inflammatory parameters in this rat model. A recent study shows that different CB2 receptor agonist has an anti-inflammatory effect in human periodontal fibroblasts [41].

A possibility of CB1-independent mechanisms in the biological effects of Meth-AEA cannot be excluded. A study using CB1 receptor knockout mice shows that Meth-AEA exerts physiological effect even in this mice model, which suggests the existence of CB1-independent effects of Meth-AEA [42]. The most investigated CB1-independent effect of Meth-AEA is its ability to activate the transient receptor potential vanilloid type-1 receptor (TRPV1). A contribution of this mechanism into the anti-inflammatory effect of Meth-AEA cannot be neglected because the ablation of TRPV1 in mice results in exacerbated immune response [43]. However, there are also some CB1-independent and TRPV1 independent effects of Meth-AEA [44]. Particularly, Meth-AEA is shown to interact with the muscarinic acetylcholine receptors [45]. Nevertheless, the role of CB1-independent mechanisms in the effect of Meth-AEA in hPdLCs is not known and must be further clarified.

The EC system can be speculated to mediate a link between stress and periodontitis. Stress is a significant risk factor influencing the immune system and potentially contributing to the progression of periodontitis [46, 47]. The EC system plays an essential role in the stress response [48]. Recently, the EC system is suggested as an important part of the neuroimmunoendocrine response in periodontal disease [49]. Our data, as well as data of previous studies [11, 14, 15, 18, 19], suggest that the EC system influences the inflammatory response in periodontitis. Emotional stress is associated with increased salivary levels of IL-6 and IL-8 [50]. Acute psychological stress is associated with the decreased tissue content of AEA in the brain, which is presumably due to an increased AEA hydrolysis [51]. It can be hypothesized that stress can also influence the levels of endocannabinoids in periodontal tissues and thus modulate their effect on the inflammatory response. However, the exact role of the EC system in the association between stress and periodontal disease should be further investigated.

The limitations of our study originate mainly from the in vitro design. We have used only one cell type and *P. gingivalis* LPS as a single inflammatory stimuli. Situation in vivo is markedly complicated and includes the interaction of several cell types in the presence of numerous inflammatory mediators and bacterial stimuli. The number of donors is rather small, and all donors were young, and therefore our results could not be representative for the whole population. We have used the cells isolated from the third molar, which also restrains the clinical translation because of the limited involvement of these teeth into masticatory function.

## Conclusion

Summarizing, our results on Meth-AEA provide further evidence of the involvement of the EC system into the progression of periodontal disease. Our data are in line with a study on rats showing that Meth-AEA reduces the clinical parameters in LPS-induced periodontitis [25]. Similarly, a local injection of AEA reduced ligature induced periodontitis in rats [19]. An involvement of the EC system in periodontitis is also suggested by studies on cannabinoid receptor expression in periodontal tissue. An in vivo study on human periodontal biopsies shows that periodontitis is associated with a decreased expression of CB1 receptor and an increased expression of CB2 receptor [11]. Another study shows an increased expression of CB1 and CB2 receptors in inflamed gingival tissue [14]. All these data suggest that the EC system plays an essential role in periodontitis.

## Data Availability

The datasets used and/or analyzed during the current study are available from the corresponding author on reasonable request.

## Abbreviations

LPS: Lipopolysaccharide
Meth-AEA: Methanandamide
hPdLCs: Human periodontal ligament cells
IL: Interleukin
MCP: Monocyte chemoattractant protein
AEA: Anandamide
2-AG: 2-arachidonoylglycerol
EC: Endocannabinoid
CB1: Cannabinoid receptor type 1
CB2: Cannabinoid receptor type 2
DMEM: Dulbecco’s modified Eagle’s medium
FBS: Fetal bovine serum
sCD14: Soluble CD14
